# Comparative Analysis of L-Carnitine and Coenzyme Q_10_ Adverse Reaction Reports Using the EudraVigilance Database: Implications for Health and Sports Supplementation

**DOI:** 10.3390/nu18111716

**Published:** 2026-05-27

**Authors:** Debora Di Mauro, Fabrizio Calapai, Ilaria Ammendolia, Mariaconcetta Currò, Fabio Trimarchi, Carmen Mannucci

**Affiliations:** 1 Department of Biomedical, Dental Sciences and Morphological and Functional Imaging, University of Messina, 98125 Messina, Italy; ddmauro@unime.it (D.D.M.); fabrizio.calapai@unime.it (F.C.); fatrim@unime.it (F.T.); 2 Department of Chemical, Biological, Pharmaceutical and Environmental Sciences, University of Messina, 98166 Messina, Italy; ilaria.ammendolia@unime.it; 3 Department of Clinical and Experimental Medicine, University of Messina, 98125 Messina, Italy; mariaconcetta.curro@unime.it

**Keywords:** L-carnitine, Coenzyme Q_10_, sports supplementation, supplements safety, individual cases safety reports

## Abstract

**Background/Objectives**: L-carnitine and Coenzyme Q_10_ (CoQ_10_) are widely used in health and sports supplementation settings to improve energy metabolism, reduce fatigue, and support recovery. Although generally perceived as safe, their safety profiles are mainly based on pre-marketing studies and selected clinical populations, while real-world pharmacovigilance evidence remains limited. This study aimed to evaluate and compare the adverse drug reaction (ADR) reporting patterns associated with L-carnitine and CoQ10 using the EudraVigilance database. **Methods**: A retrospective pharmacovigilance analysis was conducted using spontaneous individual case safety reports (ICSRs) retrieved from the EudraVigilance database. ADRs associated with L-carnitine and CoQ_10_ were analyzed and compared at the System Organ Class (SOC) level. Disproportionality analyses were performed using the reporting odds ratio (ROR) and proportional reporting ratio (PRR). **Results**: A total of 257 ICSRs for L-carnitine and 271 for CoQ10 were identified. Serious cases accounted for 34.2% of L-carnitine reports and 74.5% of CoQ_10_ reports. For L-carnitine, the most frequently reported SOC categories were gastrointestinal disorders, skin and subcutaneous tissue disorders, general disorders and administration site conditions, and nervous system disorders. For CoQ_10_, the most commonly reported SOC categories were general disorders and administration site conditions, nervous system disorders, investigations, and gastrointestinal disorders. Comparative disproportionality analysis showed higher reporting frequencies for CoQ_10_ in blood and lymphatic system disorders (ROR 3.04; PRR 2.99), musculoskeletal and connective tissue disorders (ROR 2.63; PRR 2.52). **Conclusions**: Real-world pharmacovigilance data suggest partially different ADR reporting patterns for L-carnitine and CoQ_10_ compared with those described in pre-marketing studies. CoQ_10_ was associated with a higher proportion of serious reports and greater disproportionality signals for selected SOC categories; however, these findings should be interpreted cautiously, as reporting patterns may be influenced by reporting bias, comorbidities, concomitant therapies, and differences in the populations using these compounds. Continuous pharmacovigilance monitoring and periodic reassessment of their benefit–risk profile remain essential given their widespread use in health and sports supplementation settings.

## 1. Introduction

L-carnitine is a quaternary ammonium cation, either synthesized in metabolically active organs such as the kidney, liver and brain or can be assimilated through specific foods; the best sources are animal products like meat, fish, poultry, and milk [1]. With the involvement of vitamin C, vitamin B6, niacin, and iron, carnitine is primarily synthesized in the body starting from the amino acids lysine and methionine [2]. Coenzyme Q_10_ (ubiquinone or ubidecarenone) (CoQ_10_) is used in sports to boost energy metabolism, reduce oxidative stress, and enhance recovery, typically at dosages of 30–300 mg daily [3]. Studies indicate it may improve power output, increase VO2 max, and lower markers of muscle damage like creatine kinase (CK) [4]. The ability of L-carnitine to carry long-chain fatty acids into the mitochondria, the cell’s powerhouse where they are oxidized to make energy, is its most well-known property. During times of elevated energy demand, such as exercise or fasting, this process is especially important [5]. L-carnitine is indicated for the treatment of primary and secondary carnitine deficiency and is used by athletes. L-carnitine is used in sports primarily to enhance endurance, improve fat metabolism, and speed up recovery by reducing exercise-induced muscle damage and soreness. It has also been widely investigated in relation to exercise performance and recovery, suggesting a potential effect depending on exercise intensity and training status [6,7,8].

The use of L-carnitine in sport is considered an off-label use [9]. The frequency of adverse reactions to L-carnitine is reported to be very rare (<1/1000). According to the SOC level, they are described in descending frequency order as follows: gastrointestinal disorders (vomiting, nausea, diarrhea, abdominal cramp), general disorders and administration site conditions (body odor), investigations (International Normalized Ratio increased) [10]. However, this safety profile is essentially based on pre-registration studies derived from administration in people with carnitine deficiency. Previous studies conducted in athletes and physically active individuals generally reported good tolerability and a low incidence of adverse effects under controlled experimental conditions. However, most available evidence derives from relatively small clinical or supplementation studies primarily focused on efficacy outcomes rather than systematic safety assessment [11]. Moreover, previous studies have mainly evaluated these compounds separately and under controlled experimental conditions, while comparative real-world pharmacovigilance analyses based on spontaneous reporting systems are currently lacking.

Consequently, real-world pharmacovigilance data regarding adverse reaction reporting patterns associated with these compounds remain limited.

CoQ_10_ (ubiquinone or ubidecarenone) is indicated in cases of proven CoQ_10_ deficiency, as adjuvant therapy to alleviate the symptoms of chronic heart failure, as indicated in the Summary of Product Characteristics [12]. CoQ_10_ has garnered attention for its antioxidant and anti-fatigue properties. It is an endogenous, lipophilic, vitamin-like molecule, involved in the mitochondrial respiratory chain; furthermore, it acts as an electron carrier [13,14]. After administration, in the blood, the oxidized form ubiquinone is reduced to the antioxidant ubiquinol. CoQ_10_ has a protective function against the peroxidation of phospholipids, mitochondrial membrane proteins, and deoxyribonucleic acid (DNA) [15]. It is used in athletes to enhance energy metabolism (ATP production), reduce exercise-induced oxidative stress, and improve recovery [16,17]. The use of CoQ_10_ in sports is also generally considered an off-label use.

Undesirable effects reported in the summary of product characteristics, associated with its approval, include gastrointestinal symptoms, headache, dizziness and cutaneous reactions. According to the system organ class (SOC) level, they are described in the summary of product characteristics, in descending frequency order, as follows: nervous system disorders (headache, dizziness), gastrointestinal disorders (nausea, constipation, diarrhea, pain in the stomach area, indigestion), skin and subcutaneous tissue disorders (rash, itching) [12].

Although both L-carnitine and CoQ10 are generally considered safe and are widely used off-label in sports and non-clinical settings, their safety profiles are primarily based on pre-marketing clinical studies and selected patient populations. Post-marketing pharmacovigilance evidence on their adverse reaction reporting patterns remains limited, particularly in comparative analyses using spontaneous reporting systems. Moreover, previous studies have mainly evaluated these compounds separately and under controlled experimental conditions. To our knowledge, no study has directly compared adverse reaction signals associated with L-carnitine and CoQ10 using disproportionality analysis of EudraVigilance data.

We hypothesized that real-world adverse reaction patterns associated with L-carnitine and CoQ10 may differ from those reported in pre-authorization studies and that disproportionality analysis could reveal differences between their safety profiles.

Consequently, the aim of this study was to investigate and compare the safety profiles of the two substances using spontaneous adverse drug reaction reports from the EudraVigilance database.

## 2. Methods

### 2.1. Experimental Approach

EudraVigilance is the European pharmacovigilance database managed by the European Medicines Agency (EMA), containing reports of suspected adverse reactions (SARs) associated with medicines authorized in the European Economic Area (EEA) or investigated in clinical trials. Reports are submitted by national regulatory authorities and marketing authorization holders in the form of Individual Case Safety Reports (ICSRs).

The present study was designed as an observational retrospective pharmacovigilance analysis based on secondary data retrieved from the public version of the EudraVigilance database. EudraVigilance was selected because it represents one of the largest European spontaneous reporting systems and allows the evaluation of real-world adverse reaction reporting patterns as well as the identification of potential safety signals through disproportionality analysis.

The database collects reports of suspected adverse reactions, defined as medical events temporally associated with the use of a medicinal product but not necessarily causally related to it [18].

### 2.2. Sample Data

In this observational retrospective study, CSRs reporting suspected adverse reactions associated with L-carnitine or CoQ10 and entered into the EudraVigilance database up to 31 December 2025 were included in the analysis. Data extraction from the public EudraVigilance database was subsequently performed on 10 February 2026.

The following search terms were used: L-carnitine, levocarnitine, CoQ10, ubiquinone, and ubidecarenone. The public version of the EudraVigilance database was used for data collection.

Inclusion criteria comprised serious and non-serious SARs, reports involving all age groups (from 0 to >85 years), and both sexes. Cases with incomplete demographic information were not excluded if information regarding the suspected substance and adverse reaction was available.

For each ICSR, information regarding patient characteristics (age and sex), reported adverse reactions, seriousness, and qualification of the primary source was collected. The terms “sex” and “gender” are used interchangeably here because only the variable “sex” is available within EudraVigilance; therefore, the collected information refers to biological sex [19].

Duplicate ICSRs were identified through comparison of demographic characteristics, reported adverse reactions, suspected medicinal products, concomitant therapies, and EudraVigilance local report numbers. When duplicate reports were identified, only one ICSR was retained for analysis.

### 2.3. Procedures

The public version of the EudraVigilance database was used and collection of data on SARs. For all cases, information was provided on patient characteristics (age and sex), type of adverse reaction (often more than one for each ICSR), and qualification of the primary source.

The analyzed ICRs came from both healthcare and non-healthcare personnel. Regarding the data selection criteria, in ICSRs, SARs selection was based on the Medical Dictionary for Regulatory Activities (MedDRA). MedDRA is an international standardized and clinically validated medical terminology used by regulatory authorities and the biopharmaceutical industry. It is used to code cases of adverse effects in pharmacovigilance databases and to facilitate searches in the databases on adverse drug reactions. For the present study, each ICSR was analyzed and every reported SAR was extracted and counted from every single case. Single adverse reactions were described using the so-called “Preferred terms” (PTs) listed in MedDRA.

MedDRA PTs retrieved from EudraVigilance, were reviewed individually and aggregated into clinically meaningful composite categories to facilitate the interpretation of pharmacovigilance signals. PT grouping was performed using predefined criteria based on shared clinical manifestations and pathophysiological similarity. Each PT was assigned to a single composite category to avoid duplicate counting. SOC allocation followed MedDRA primary SOC classifications.

A PT is a distinct descriptor (single medical concept) for an adverse symptom or sign. We selected all the PTs that were recorded in each ICSR, we counted them all and analyzed the frequencies for every adverse reaction. Two or more PTs with overlapping clinical meanings were aggregated to avoid unnecessary duplicate PTs with the same connotation. MedDRA has a hierarchy of terms to describe adverse reactions. Adverse reactions were also grouped under the terms of the SOC (System Organ Class) level in the MedDRA hierarchy, such as for example “Musculoskeletal and connective tissue disorders”, “Vascular disorders”, etc. Each single PT has been associated with the corresponding SOC level by using the MedDRA terminology reported by the National Center for Biomedical Ontology (NCBO). The SOC system organ classification is the highest level of the hierarchy that captures the broadest concept useful for retrieving data. It is a way of grouping medical terms based on body systems or functions [20]. The complete mapping of MedDRA Preferred Terms (PTs), composite categories, and corresponding System Organ Classes (SOCs) is reported in Supplementary Table S1.

### 2.4. Statistics

A disproportionality analysis of adverse reactions aggregated according to the SOC level was performed by calculation of the reporting odds ratio (ROR) and proportional reporting ratio (PRR) comparing data of signals of spontaneous reports related to the intake of L-carnitine and CoQ_10_. The Reporting Odds Ratio (ROR) is a disproportionality measure used to identify safety signals through comparison of the odds of a specific adverse event signaled to be linked to the use of a particular drug versus the odds of the same adverse event signaled with other drugs [21]. The Reporting Odds Ratio (ROR) was calculated as (a/c)/(b/d), where a represents the number of reports for a specific adverse event with the drug of interest, b the number of reports for the same event with all other drugs, c the number of all other events with the drug of interest, and d the number of all other events with all other drugs. The Proportional Reporting Ratio (PRR) was calculated as [a/(a + c)]/[b/(b + d)].

The PRR is a disproportionality measure useful for identifying whether a drug-event combination is reported at a higher-than-expected rate relative to other products [20].

In the present analysis, disproportionality signals were considered potentially relevant when the lower limit of the 95% confidence interval (CI) of the ROR was greater than 1, and PRR values, indicating a higher-than-expected reporting frequency for a specific SOC category. However, these measures should be interpreted as indicators of reporting disproportionality rather than evidence of causality or incidence risk [20,21]. 

Data extraction was performed using a structured line-listing table in which each row represented an Individual Case Safety Report (ICSR) and each column corresponded to a specific data element associated with that case. Data were analyzed by aggregating the PTs from individual reports to higher levels of the MedDRA hierarchy by grouping individual suspected adverse reactions (SARs) at the System Organ Class (SOC) level. (e.g., nausea and vomiting classified in the same group as Gastrointestinal Symptoms). Adequate stratification of signals by sex groups was performed to avoid biases caused by confounding effects and to analyze this variable separately. Duplicate ICSRs were excluded from the analysis. Duplicate search was based on detection in the dataset of similarities in adverse reaction, age, sex, suspected/interacting medicinal products, EudraVigilance local report number. Chi-square tests were used to assess sex-based differences in categorical variables, as they are appropriate for comparing proportions between independent groups in large spontaneous reporting datasets. Given the exploratory nature of pharmacovigilance disproportionality analyses, no formal adjustment for multiple comparisons was applied. Results were interpreted as signal detection measures rather than confirmatory statistical inferences.

A descriptive statistical analysis was performed using SPSS statistical software, version 29.0 (SPSS, IBM, Armonk, NY, USA).

## 3. Results

ICSRs reporting suspected adverse reactions to L-carnitine in EudraVigilance were 257. Serious/non-serious cases ratio for L-carnitine was 0.52 and the percentage of serious cases was 34.2% of the total number of signals. ICSRs reporting suspected adverse reactions to CoQ_10_ found in EudraVigilance were 271. Serious/non-serious cases ratio for CoQ_10_ was 2.93 and the percentage of serious cases was 74.5% of the total number of signals of adverse reactions to CoQ_10_ (Table 1).

**Figure 1:**
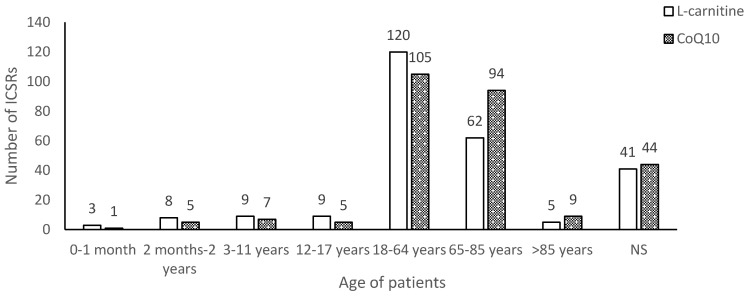
Individual Cases Safety Reports (ICSRs) reporting suspected adverse reactions to L-carnitine and CoQ_10_, stratified by patient age.

Individual cases with SARs to L-carnitine were distributed according to their age as follows: 0–1 month (3), 1.2%, 2 months–2 years (8) 3.1%, 3–11 years (9) 3.5%, 12–17 years (9) 3.5%, 18–64 years (120) 46.7%, 65–85 years (62) 24.1%, more than 85 years (5) 1.19% and not specified (41) 15.9% (Figure 1). Sex distribution showed the apparent higher proportion of reports in females (61.5%). Most frequently reported adverse reactions, according to the analysis of the frequency of PTs, were in decreasing order: dizziness/vertigo, abdominal discomfort/abdominal pain, urticaria/pruritus, nausea, erythema, agitation, diarrhea, paraesthesia, vomiting, and tremor. However, statistically significant female predominance was observed. only for the adverse reaction “agitation” (Table 2).

Although a higher absolute number of reports was observed in females, statistically significant sex differences were identified only for agitation among L-carnitine-related reports.

ICSRs with SARs to CoQ_10_ were distributed according to their age as follows: 0–1 month (1), 0.4%, 2 months–2 years (5) 1.8%, 3–11 years (7) 2.6%, 12–17 years (5) 1.8%, 18–64 years (105) 38.7%, 65–85 years (94) 34.7%, more than 85 years (9) 3.3% and not specified (44) 16.2% (Figure 1). Sex distribution showed the prevalence of signals of adverse reactions occurring in females (61.6%). Most frequently signaled adverse reactions to CoQ_10_, according to the analysis of the frequency of PTs, were in decreasing order: abdominal discomfort/abdominal pain, nausea, arthralgia/arthritis, rash, acute hepatic failure/hepatitis, anaemia, and decreased blood pressure. Analysis of sex distribution for individual adverse reactions associated with L-carnitine showed an apparent prevalence of signals regarding females (61.6%) over males (Table 2).

No significant difference was shown in the sex distribution of single adverse reactions to CoQ_10_ (Table 3).

After aggregation of the adverse reactions according to the SOC level the results of the disproportionality test, calculated as ROR and PRR, showed that the use of L-carnitine was associated with spontaneous reports of adverse reactions regarding gastrointestinal disorders (14.6%), skin and subcutaneous tissue disorders (14.0%), general disorders and administration site conditions (13.75%), nervous system disorders (12.44%). Groups of most frequent adverse reactions to CoQ_10_ were general disorders and administration site conditions (13.07%), nervous system disorders (12.83%), investigations (9.59%), gastrointestinal disorders (9.46%) (Table 4). Table 4 summarizes the disproportionality analysis, expressed as reporting odds ratio (ROR) and proportional reporting ratio (PRR), comparing CoQ10 versus L-carnitine. Higher reporting frequencies for blood and lymphatic system disorders and musculoskeletal and connective tissue disorders were observed with CoQ10 compared with L-carnitine (Table 4).

## 4. Discussion

The present study aimed to compare adverse reaction reporting patterns associated with L-carnitine and CoQ10 using real-world pharmacovigilance data retrieved from the EudraVigilance database. Although the overall number of Individual Case Safety Reports (ICSRs) was comparable between the two compounds, important differences emerged in the reporting patterns. In particular, CoQ10 was associated with a higher proportion of serious reports and with higher disproportionality signals in selected System Organ Classes (SOCs), including blood and lymphatic system disorders, musculoskeletal and connective tissue disorders. These findings suggest partially different pharmacovigilance reporting profiles between the two substances in real-world settings.

However, these results should be interpreted cautiously. Spontaneous reporting systems such as EudraVigilance are inherently affected by several limitations, including underreporting, reporting bias, incomplete clinical information, and the inability to establish causal relationships or incidence rates [20,21,22,23]. Consequently, disproportionality analyses identify reporting patterns rather than confirmed safety risks and should therefore be considered hypothesis-generating tools.

The higher proportion of serious reports observed for CoQ10 does not necessarily indicate a greater intrinsic toxicity of the compound. A possible explanation is related to differences in the characteristics of the populations using these supplements in real-world settings. CoQ10 is frequently used not only in sports supplementation but also in older individuals and patients affected by cardiovascular, metabolic, or chronic diseases, often receiving multiple concomitant therapies. These clinical conditions may independently increase the likelihood of serious clinical outcomes and consequently influence reporting behavior. In contrast, L-carnitine is frequently used in younger or physically active populations and may therefore be associated with different reporting dynamics. Furthermore, serious or clinically relevant adverse events are generally more likely to be reported to pharmacovigilance systems than mild or expected reactions, potentially contributing to disproportionality signals.

The analysis of adverse reactions aggregated at the SOC level showed that gastrointestinal disorders, skin and subcutaneous tissue disorders, general disorders and administration site conditions, and nervous system disorders were among the most frequently reported categories for L-carnitine. These findings are generally consistent with previous clinical and pharmacological literature describing gastrointestinal symptoms, mild neurological manifestations, and cutaneous reactions among the most commonly reported adverse effects associated with L-carnitine supplementation [10,11,24,25,26,27,28,29]. Dizziness and vertigo, which were represented among the most frequently reported Preferred Terms (PTs) in the present analysis, have occasionally been described in clinical studies and case reports, although they are not consistently emphasized in pre-marketing safety summaries. This discrepancy may reflect the broader heterogeneity of populations included in spontaneous reporting systems compared with controlled clinical studies.

For CoQ10, the most frequently reported SOC categories included general disorders and administration site conditions, investigations, and gastrointestinal disorders. Previous studies and systematic reviews have generally described CoQ10 as well tolerated both in healthy individuals and in athletes, with gastrointestinal symptoms, headache, dizziness, and mild cutaneous reactions representing the most commonly reported adverse events [4,12,15,16,30]. Nevertheless, the present pharmacovigilance analysis identified higher disproportionality signals for selected SOC categories compared with L-carnitine. These findings may be influenced by the characteristics of real-world users, concomitant therapies, and the broader clinical complexity of patients exposed to CoQ10 outside controlled experimental settings.

A relevant aspect in the interpretation of CoQ10-related safety signals concerns its potential interaction with anticoagulant therapies, particularly warfarin. Previous literature has suggested that CoQ10 may interfere with anticoagulation control because of its structural similarity to vitamin K, potentially affecting coagulation parameters in susceptible individuals [31]. Although the present analysis identified disproportionality signals involving blood and lymphatic system disorders, the publicly accessible EudraVigilance dataset did not consistently provide sufficient clinical detail to systematically evaluate confirmed drug–drug interactions, concomitant therapies, or causality assessment. Therefore, no definitive conclusions regarding interaction-related adverse reactions can be drawn from the present data.

The present study has several strengths. First, it provides a comparative pharmacovigilance evaluation of two widely used supplements using one of the largest European spontaneous reporting systems. Second, the study contributes real-world evidence regarding adverse reaction reporting patterns associated with compounds frequently used in both clinical and sports supplementation settings. Third, the use of disproportionality analysis allowed the identification of differential reporting signals that may deserve further investigation in future pharmacoepidemiological studies.

Nevertheless, several important limitations should be acknowledged. Underreporting remains a major limitation of spontaneous reporting systems because only a fraction of adverse reactions are reported. In addition, the absence of reliable exposure denominators prevents estimation of incidence rates or absolute risks. The quality and completeness of ICSRs may also vary substantially, with incomplete information regarding comorbidities, concomitant medications, dosages, duration of exposure, and clinical outcomes. Moreover, the EudraVigilance database does not systematically distinguish between use in sports supplementation and clinical therapeutic use. Reporting behavior may additionally be influenced by media attention, regulatory warnings, publication bias, or differences in healthcare professional awareness. Consequently, disproportionality measures such as ROR and PRR should not be interpreted as indicators of causality or comparative toxicity, but rather as tools for signal detection requiring further clinical confirmation.

In conclusion, the present real-world pharmacovigilance analysis suggests that L-carnitine and CoQ10 exhibit partially different adverse reaction reporting patterns in the EudraVigilance database. CoQ10 was associated with a higher proportion of serious reports and higher disproportionality signals in selected SOC categories compared with L-carnitine. However, these findings should be interpreted within the context of the inherent limitations of spontaneous reporting systems and should be considered hypothesis-generating rather than conclusive evidence of differential safety profiles. Continuous pharmacovigilance monitoring and further real-world studies are warranted to better characterize the safety profiles of supplements widely used in both clinical practice and sports settings.

## Figures and Tables

**Table 1:** Serious/non-serious ratio of Individual Cases Safety Reports (ICSRs) related to L-carnitine and CoQ_10_ signaled in the European Economic Area and United Kingdom.

Drug	ICSRs (Total)	Serious ICSRs	Non-Serious ICSRs	ICSRs Serious/Non-Serious Ratio
CoQ10	271	202	69	2.93
L-Carnitine	257	88	169	0.52

ICRs: Individual Cases Safety Reports.

**Table 2:** Frequency and sex distribution of cases of suspected adverse reactions (SARs) to L-carnitine in the European Economic Area and the United Kingdom.

SARs	Females Cases = 158	Males Cases = 99	Total Cases	Chi Square Statistic	*p* Value
Dizziness/Vertigo	17	9	26	0.1864	0.665969
Abdominal discomfort/ Abdominal pain	19	5	24	3.4972	0.061472
Urticaria/Pruritus	12	8	20	0.0200	0.887478
Nausea	13	5	18	0.9434	0.331412
Erythema	8	4	12	0.1431	0.705253
Agitation	10	1	11	4.2030	0.040352 *
Diarrhea	4	7	11	3.0608	0.080204
Paraesthesia	8	3	11	0.6140	0.433286
Vomiting	8	2	10	1.5072	0.219574
Tremor	5	5	10	0.5789	0.446752
Rash	5	4	9	0.1382	0.710115
Headache	7	1	8	2.3608	0.124418
Insomnia	6	2	8	0.6374	0.424638
Tachycardia/Palpitations	4	4	8	0.4594	0.497912
Dyspepsia	4	3	7	0.0571	0.811106
Hypertension	3	3	6	0.3418	0.558796
Confusional state	2	3	5	0.9933	0.318933
Dyspnoea	4	1	5	0.7386	0.390102
Hallucination	4	1	5	0.7386	0.390102

SARs: Suspected adverse reactions. Only adverse reactions signaled more than four times are reported. * = *p* < 0.05.

**Table 3:** Frequency and sex distribution of cases of suspected adverse reactions (SARs) to CoQ_10_ in the European Economic Area and the United Kingdom.

SARs	Females Cases = 167	Males Cases = 104	Total Cases	Chi Square Statistic	*p* Value
Abdominal discomfort/ Abdominal pain	24	9	33	1.959	0.161624
Nausea	3	4	7	0.9434	0.331412
Arthralgia/arthritis	6	1	7	1.0701	0.300927
Rash	2	4	6	2.0765	0.149580
Acute hepatic failure/Hepatitis	2	3	5	1.0072	0.315582
Anaemia	2	3	5	1.0072	0.315582
Blood pressure decreased	2	3	5	1.0072	0.315582

SARs: Suspected adverse reactions. Only adverse reactions signaled more than four times are reported.

**Table 4:** Disproportionality analysis of adverse reaction reports associated with CoQ10 versus L-carnitine at the System Organ Class (SOC) level.

SOC	SARs to CoQ_10_ N. and (%)	All Other SARs to CoQ_10_	SARs to Carnitine N. and (%)	All Other SARs to Carnitine	ROR CoQ_10_ vs. Carnitine (95% C.I.)	PRR
Blood and lymphatic system disorders	21 (2.61%)	782	4 (0.87%)	454	**3.04** **(1.04–8.93)**	**2.99**
Cardiac disorders	30 (3.73%)	773	18 (3.93%)	440	0.69 (0.39–1.22)	0.70
Ear and labyrinth disorders	14 (1.74%)	789	9 (1.96%)	449	0.88 (0.38–2.06)	0.89
Eye disorders	21 (2.61%)	782	9 (1.96%)	449	1.34 (0.61–2.95)	1.33
Gastrointestinal disorders	76 (9.46%)	727	67 (14.6%)	391	0.61 (0.43–0.87)	0.65
General disorders and administration site conditions	105 (13.07%)	698	63 (13.75%)	395	0.94 (0.67–1.32)	0.95
Immune system disorders	11 (1.37%)	792	7 (1.53%)	451	0.89 (0.34–2.32)	0.90
Infections and infestations	35 (4.36%)	768	10 (2.18%)	448	2.04 (1.00–4.16)	2.00
Injury, poisoning and procedural complications	53 (6.60%)	750	29 (6.33%)	429	1.04 (0.65–1.67)	1.04
Investigations	77 (9.59%)	726	21 (4.58%)	437	**2.21** **(1.34–3.63)**	**2.09**
Metabolism and nutrition disorders	25 (3.11%)	778	16 (3.49%)	442	0.89 (0.47–1.67)	PRR: 0.89
Musculoskeletal and connective tissue disorders	53 (6.60%)	750	12 (2.62%)	446	**2.63** **(1.34–4.97)**	**2.52**
Nervous system disorders	103 (12.83%)	700	57 (12.44%)	401	1.04 (0.73–1.46)	1.03
Psychiatric disorders	41 (5.10%)	762	34 (7.42%)	424	0.67 (0.42–1.07)	0.69
Renal and urinary disorders	14 (1.74%)	789	14 (3.06%)	444	0.56 (0.26–1.19)	0.57
Reproductive system and breast disorders	13 (1.62%)	790	3 (0.65%)	455	2.49 (0.71–8.80)	2.47
Respiratory, thoracic and mediastinal disorders	31 (3.86%)	772	15 (3.27%)	443	1.18 (0.63–2.22)	1.18
Skin and subcutaneous tissue disorders	54 (6.72%)	749	64 (14.00%)	394	0.44 (0.30–0.65)	0.48
Vascular disorders	26 (3.24%)	777	9 (1.96%)	449	1.67 (0.77–3.59)	1.65

SOC: System Organ Class; ROR: Reporting Odds Ratio; PRR: Proportional reporting ratio; SARs: suspect adverse reactions; C.I. = confidence intervals. ROR values > 1 indicate higher reporting Frequency with CoQ10 compare with L-carnitine.

## Data Availability

The data analyzed and presented in this study are available on the public EudraVigilance database, https://www.ema.europa.eu/en/human-regulatory-overview/research-development/pharmacovigilance-research-development/eudravigilance (accessed on 10 February 2026).

## Supplementary Materials

The following supporting information can be downloaded at: https://www.mdpi.com/article/10.3390/nu18111716/s1. Table S1. Mapping of MedDRA Preferred Terms (PTs) into composite categories and corresponding System Organ Classes (SOCs).
