# Supplementary material for: Comparative Analysis of L-Carnitine and Coenzyme Q10 Adverse Reaction Reports Using the EudraVigilance Database: Implications for Health and Sports Supplementation
Source: Nutrients. 2026 May 27;18(11):1716. doi: 10.3390/nu18111716 (PMC13258800; doi:10.3390/nu18111716)
Supplement: Supplementary file 1 [file nutrients-18-01716-s001.zip › nutrients-4329929-supplementary.pdf]

Supplementary Table S1. Mapping of MedDRA Preferred Terms (PTs) into composite categories and corresponding System Organ Classes (SOCs).

Preferred Term (PT)	Composite Category	System Organ Class (SOC)
Nausea	Gastrointestinal symptoms	Gastrointestinal disorders
Vomiting	Gastrointestinal symptoms	Gastrointestinal disorders
Diarrhoea	Gastrointestinal symptoms	Gastrointestinal disorders
Abdominal discomfort	Gastrointestinal symptoms	Gastrointestinal disorders
Abdominal pain	Gastrointestinal symptoms	Gastrointestinal disorders
Dyspepsia	Gastrointestinal symptoms	Gastrointestinal disorders
Dizziness	Neurological symptoms	Nervous system disorders
Vertigo	Neurological symptoms	Nervous system disorders
Tremor	Neurological symptoms	Nervous system disorders
Paraesthesia	Neurological symptoms	Nervous system disorders
Headache	Neurological symptoms	Nervous system disorders
Confusional state	Neuropsychiatric symptoms	Nervous system disorders
Agitation	Psychiatric symptoms	Psychiatric disorders
Hallucination	Psychiatric symptoms	Psychiatric disorders
Insomnia	Psychiatric symptoms	Psychiatric disorders
Urticaria	Cutaneous hypersensitivity reactions	Skin and subcutaneous tissue disorders
Pruritus	Cutaneous hypersensitivity reactions	Skin and subcutaneous tissue disorders
Rash	Cutaneous hypersensitivity reactions	Skin and subcutaneous tissue disorders
Erythema	Cutaneous hypersensitivity reactions	Skin and subcutaneous tissue disorders
Arthralgia	Musculoskeletal symptoms	Musculoskeletal and connective tissue disorders
Arthritis	Musculoskeletal symptoms	Musculoskeletal and connective tissue disorders
Dyspnoea	Respiratory symptoms	Respiratory, thoracic and mediastinal disorders
Tachycardia	Cardiovascular symptoms	Cardiac disorders
Palpitations	Cardiovascular symptoms	Cardiac disorders
Hypertension	Vascular symptoms	Vascular disorders
Blood pressure decreased	Vascular symptoms	Vascular disorders
Anaemia	Hematological disorders	Blood and lymphatic system disorders
Acute hepatic failure	Hepatic disorders	Hepatobiliary disorders
Hepatitis	Hepatic disorders	Hepatobiliary disorders
